# COVID-19 and surgery: Running on good will or guilt?

**DOI:** 10.1016/j.amsu.2020.05.019

**Published:** 2020-05-20

**Authors:** Jaideep Singh Rait, Charannya Balakumar, Pierre Montauban, Prizzi Zarsadias, Sara Iqbal, Ankur Shah

**Affiliations:** William Harvey Hospital, Kennington Road, Ashford, Kent, TN23 0LZ, UK

To the editor:

The recent pandemic crisis engulfing the NHS has placed an increased demand on the healthcare workforce with unprecedented numbers of clinicians being recalled to service, medical students being accelerated through their training and the redeployment of clinicians to areas requiring increased support.

Within surgery current guidelines and advice have been in flux since the start of this crisis and as such there has been much confusion, speculation and anxiety associated with each new day. Many of our colleagues have been affected directly by COVID-19 with many being forced into isolation whether symptomatic or asymptomatic.

The overriding feeling of powerlessness in the face of this virus amongst surgeons has been invading the consciousness of us all. Many of our colleagues have felt guilt and associated frustration when forced to isolate despite feeling well and leaving our colleagues to pick up the strain. Equally, those surgeons currently unaffected are being placed under new stress when trying to deal with the challenges associated with this pandemic.

There is a pervading feeling of guilt amongst many surgeons. The feeling that we can and should be doing more to assist our medical and intensivist colleagues. Many trainees are being moved to critical care areas or medicine itself, creating its own level of anxiety associated with unfamiliar surroundings and procedures at a time of crisis.

The NHS has long run on the good will of its staff, to support each other, to support each department and to cover up for shortcomings caused by lack of funds, lack of resources or lack of staffing. In the current crisis there has been an infiltration of guilt amongst many of our colleagues in isolation to the point that many have returned to service earlier than they perhaps should have, to aid their colleagues, assuage their guilt and keep the spirit of goodwill within the NHS.

Important lessons will be learnt from this time of crisis. One such lesson that must not be overlooked is when the goodwill of staff tips over to guilt and becomes something no longer to be praised but something to be wary of. Each surgeon will have their own reason for feeling guilt but this negative emotion must be combatted at all levels to prevent negative coping strategies creeping in at a time when we must all remain strong and united to triumph.

## Provenance and peer review

Not commissioned, Editor reviewed.

